# Warming Houses

**Published:** 1860-10

**Authors:** 


### WARMING HOUSES.

IN cheerful comfort there is nothing equal to a blazing wood-fire, on a commodious hearth. The very thought of it carries us backwards to days of unbridled gladness and joyous youth and genial sunshine. For purity of atmosphere and consequent healthfulness, there can be no superior to the old-fashioned fire-place, "and-irons," back-logs and fore-sticks, with the broad bed of flaming red coals!

Next to the wood fire-place, is the "Low-down grate," of recent introduction, suitable for burning every kind of fuel; wood, soft coal, anthracite, red ash, bituminous, Liverpool, Cannel, any thing. It is in reality a "fire-place;" the fuel is placed flat on the hearth, on a level with the floor, the jambs are broad and flaring, there is but little use for a poker or "blower," and hence no dust. The ashes fall through a grating into a receptacle which may be emptied daily, or are conveyed through an iron pipe into a close brick chamber in the cellar, to be removed once a year. By this contrivance the feet are easily warmed, and are kept so; there is no danger of the coals falling on the floor or carpet, and the fire is made to burn more or less fiercely as easily as in an air-tight stove. This is written after a winter's trial. At an expense of less than three tons of coal, or two hundred and forty bushels, the thermometer on the wall opposite to the fire-place, in a room two hundred and fifty feet square and twelve high, was kept at sixty-five degrees when the mercury was in the neighborhood of zero without, the heat being derived from a broad bed of glowing coals over two feet long. These coals being on a level with the floor, keep the feet delightfully warm. The air for combustion is obtained from the cellar or the street; hence the atmosphere of the room is simply pure air warmed, and has the genial heat of a wood-fire; hence, also, there is none of the feeling of heaviness, sultriness, and oppression which is instantly experienced on entering a furnace or stove-heated apartment. We certainly feel that the perfection of house-warming in our country at present is to have a low-down grate in each sitting apartment, while the extra heat is economized, to be thrown into chambers, sufficient to take off the chilliness or dampness when retiring or rising in the coldest weather. If families are so constituted that there must be additional heat, at least in cases of sickness, or company, or extra severe weather, when it may be desirable to modify the atmosphere of the halls between the temperature of out-doors and that of the sitting rooms, Bartlett's Portable Furnace answers the purpose most admirably, which, by being placed in the lower hall, and being so contrived that the warm air given out can not come in contact with red-hot iron, supplies an atmosphere for breathing which is pure and exhilarating. Such was our practice last winter, the fire being kindled in the portable furnace in the lower hall only for seven days during the whole season, and these were, not at times when the weather was the coldest, because then the air was purest, driest, and most bracing, but for the days coming after the coldest ones, when there was an ugly damp chilliness in the air, which, by abstracting the heat rapidly from the body, produced a stronger impression of coldness than when the weather was twenty degrees colder, but still and dry, for it is not in the very coldest weather, when zero is hugged by the mercury, that "colds" are so much taken, but when the air is raw from being saturated with dampness. It is in thawy weather that furnaces should be heated up, if ever. By this arrangement there was scarcely a cold in the family, varying in age from five to seventy-five, during the whole winter.

Next to a wood-fire or a low-down grate for coal, preference should be given to the method of warming houses by the "Radiator." This is the latest novelty, and is the best; its expensiveness in the first construction is perhaps the only drawback worth consideration. The fire is built in the cellar, and it is so contrived that the heat is given out in any hall or room by means of *large surfaces*, which never can become red-hot nor any thing like it, nor, indeed, is it ever necessary, even in the very coldest weather. We know at present of no pattern of furnaces but may be heated to redness by the negligence of ser vants, and which fail to keep a large house comfortably warm in very cold weather, even if they are red-hot, and this is the fatal objection to furnace heat; the surface heated is so small, that where there is great cold, red heat is a necessity, in order to give sufficient warmth; but in case of the "Radiator," the surface is so large, that it gives out an immense amount of heat to an apartment when it is itself moderately heated. It costs about a thousand dollars to introduce the "Radiator" into a common-sized dwelling, and it must consume an amount of coal equal to the common furnace, but it gives a genial and pure warmth—no dust, no explosions, no leakages. It may be "comfortable" to have a whole house heated; but in whatever way it is done in this country, except in the ways we have recommended, it is done to the injury of house, furniture, and health of the families exposed to the pernicious influences of foul gases, oppressive fumes, and an innutritious atmosphere. Incomparably better would it be to use the low-down grate altogether, with the portable furnace for the hall, only to be fired in cold, damp, raw weather, or when the thermometer is about zero for several days together. The several sizes of the low-down grate are furnished and slipped into the ordinary fire-place at a cost of from thirty to fifty dollars each.

